# Serum concentration of polychlorinated biphenyls and the risk of type 2 diabetes: a 10-year follow-up historical cohort study

**DOI:** 10.1038/s41598-024-59308-9

**Published:** 2024-04-12

**Authors:** Masoumeh Ravanipour, Iraj Nabipour, Masud Yunesian, Noushin Rastkari, Amir Hossein Mahvi

**Affiliations:** 1 Department of Environmental Health Engineering, School of Public Health, Tehran University of Medical Sciences, PourSina St., Qods St., Enghelab St., Tehran, 141761315 Iran; 2 Department of Environmental Health Engineering, Faculty of Health and Nutrition, Bushehr University of Medical Sciences, Bushehr, Iran; 3 The Persian Gulf Tropical Medicine Research Center, The Persian Gulf Biomedical Sciences Research Institute, Bushehr University of Medical Sciences, Bushehr, Iran; 4 Department of Internal Medicine, School of Medicine, Bushehr University of Medical Sciences, Bushehr, Iran; 5 Department of Research Methodology and Data Analysis, Institute for Environmental Research, Tehran University of Medical Sciences, Tehran, Iran; 6 Center for Air Pollution Research Center, Tehran University of Medical Sciences, Tehran, Iran

**Keywords:** PCBs, Persistent organic pollutants, Environmental pollutants, Diabetes mellitus, Diabetes risk, Endocrinology, Environmental monitoring, Risk factors

## Abstract

This study investigated the association between serum concentrations of Polychlorinated Biphenyls (PCBs) and the risk of type 2 diabetes within the general population. A ten-year follow-up historical cohort study was conducted during 2009–2019 as part of the Bushehr MONICA cohort study in Iran. Of 893 non-diabetes participants at base line, 181 individuals were included in the study. The concentration of nine PCB congeners was measured in individuals’ serum samples at baseline, and the risk of type 2 diabetes was determined based on fasting blood sugar at the end of follow-up. Multiple logistic regression models were used to assess the study outcomes after adjusting for covariates. This study included 59 diabetes individuals (32.6%; mean [SD] age: 58.64 [8.05]) and 122 non-diabetes individuals (67.4%; mean [SD] age: 52.75 [8.68]). Multivariable analysis revealed that a one-tertile increase (increasing from 33rd centile to 67th centile) in Σ non-dioxin-like-PCBs (OR 2.749, 95% CI 1.066–7.089), Σ dioxin-like-PCBs (OR 4.842, 95% CI 1.911–12.269), and Σ PCBs (OR 2.887, 95% CI 1.120–7.441) significantly associated with an increased risk of type 2 diabetes. The strongest association was obtained for dioxin-like PCBs. The results highlight a significant correlation between PCB exposure and an increased risk of type 2 diabetes. The evidence suggests that additional epidemiological studies are necessary to clarify the link between PCBs and diabetes.

## Introduction

Diabetes Mellitus (DM) is a prevalent non-communicable disease and a major challenging health problem in the twentieth century, characterized by increasing the concentration of blood glucose^1,2^. According to the World Health Organization (WHO), diabetes mellitus was the ninth global cause of death and the eighth global cause of disability-adjusted life years (DALYs) in 2019^3^. Over the last decades, the prevalence trend of diabetes has increased rapidly worldwide, especially in low and middle-income countries^4^. This dramatic increase in the prevalence of diabetes mellitus highlights the need to better understand the role of its risk factors. Various traditional risk factors have been reported as known factors of diabetes such as physical activity, diet, BMI/obesity, stress, smoking, alcohol consumption, metabolic syndrome, and vitamin D^5,6^. Approximately 6% of the causes of type 2 diabetes can be attributed to genetic factors^5,7^, but environmental factors may play an essential role in the prevalence of this disease^5^ along with other traditional risk factors. Recent evidence shows polychlorinated biphenyls (PCBs), an essential class of persistent organic pollutants (POPs), are among environmental pollutants that have been mentioned as probable risk factor for type 2 diabetes^5,6^. PCBs are a group of chlorinated aromatic hydrocarbons with one to ten chlorine atoms. This group includes 209 congeners, segregated into non-dioxin-like PCBs (NDL-PCBs) with 197 congeners and dioxin-like PCBs (DL-PCBs) with 12 congeners^8,9^. These substances were incorporated into the Stockholm Convention in 2001, with signatories committing to cease all production and eliminate their use and release, aiming to reduce their risks by 2025^8,10^. Despite such regulatory measures, daily PCBs exposure is possible due to their resistance in the environment, high lipid solubility, and biomagnification. Accordingly, the general population can still expose to PCBs through various direct and indirect routes, including food intake (i.e., consumption of high-fat foods such as eggs, dairy products, and animal fats), drinking water, inhalation, dermal contact etc.^11,12^. These compounds not only lead to adverse health outcomes such as cancers, immune and hormonal system disorders, central and peripheral nervous system damage, reproductive effects, and liver disorders^13,14^, but also classify as endocrine disrupting compounds^8^.

Although various experimental and epidemiological studies have investigated on the environmental exposure to PCBs and type 2 diabetes, there is still conflicting evidence regarding this association^2,15–18^. Additionally, in terms of design, fewer studies with focus on the general populations have designed longitudinally^2,19,20^, while most studies have been cross-sectional^12,21,22^. In this regard, a review study emphasized that there is poor evidence to suggest an association between PCBs and the development of diabetes due to a small number of prospective studies or inappropriate animal model studies^23^. Also, a meta-analysis highlighted the urgent need for further research into the effects of endocrine disruptor compounds on the risk of type 2 diabetes through more extensive prospective studies^24^.

Addressing these research gaps, to achieve further insight into the association between PCBs and the risk of type 2 diabetes, we investigated a historical cohort study within the Prospective Investigation of Bushehr MONICA study (PIB-MONICA-S), measuring nine PCB congeners in participants’ blood serum and following up diabetes for ten years. For this purpose, we hypothesized that there is a relationship between the serum PCB levels in individuals with type 2 diabetes and those without, aiming to illuminate PCBs’ role in the epidemiology of type 2 diabetes.

## Methods

### Source population

The Prospective Investigation of Bushehr MONICA study, a part of the WHO MONICA Project, was conducted as a longitudinal population-based cohort study in two phases from 2003 to 2009. The PIB-MONICA study aimed to determine the cardiovascular diseases risk factors within the northern Persian Gulf population. Various clinical and biological tests were performed for the participants and information was recorded. The participants’ blood serum samples were also stored at − 80 °C in a bio-bank. The sampling procedure details of the PIB-MONICA study have been extensively described elsewhere^25,26^.

### Study population

We conducted a historical cohort study utilized baseline data from phase II of the PIB-MONICA study. Participants who were in phase II of the study between 2009 and 2010 and followed up for 10 years, were invited to this study conducted between 2019 and 2020. All non-diabetic individuals, based on their registered data at the end of phase II, were identified (n = 893 out of 1092). We were informed about their condition and current use of insulin or anti-diabetic drugs through a telephone interview. All non-diabetic individuals residing in Bushehr, Iran, were eligible for this study (n = 515), out of which 70 reported having diabetes and 445 reported no diabetes based on the phone self-declaration. The remaining participants (n = 378) were excluded for various reasons, such as death, relocation, non-response due to a phone failure or changed numbers, or unwillingness to participate in the study. The number of samples was limited to the number of eligible people from the baseline who were willing to participate in this study. For calculating the study sample size, we used Codru et al.^21^, a cross sectional PCBs evaluation in diabetic adult Native-Americans, as a reference. We calculated the sample size with a power of 80%, alpha = 0.05, the odds ratio (OR) of 3.9, and the prevalence of exposure with diabetes in case group of 0.33. The planned sample size for case group was 53 and considering r = 2 (the ratio of control to case), the sample size of the control group was also calculated. Taking into consideration the 15% drop-out rate, we indicating that a total of 183 participants would be required. Since the sampling period coincided with the COVID-19 lockdown, it affected the participation rates, especially in the case group, and we could not achieve the desired number of cases. Ultimately, 181 individuals (59 people with diabetes and 122 non-diabetes) were included in the study.

Subsequently, blood samples were taken from all participants to measure some biochemical characteristics, including fasting blood sugar (FBS), total cholesterol (TC), high-density lipoprotein (HDL) cholesterol, and triglycerides (TG). Low-density lipoprotein (LDL) cholesterol levels were calculated using the Friedewald formula^27^. Additionally, other anthropometric indices such as blood pressure (measured twice after 15 min of rest in the right arm), height, weight, and waist and hip circumference were measured and recorded using a modified retrospective data collection form, which was based on the standardized WHO MONICA Project questionnaire^25,26^.

### Outcome assessment

The primary outcome, the risk of type 2 diabetes, was assessed through fasting blood sugar (FBS) serum levels. For this purpose, we considered the following definitive criteria for the participant with diabetes: having fasting blood sugar (FBS) ≥ 126 mg/dl^28–30^ or consuming anti-diabetes drugs.

### Covariates

Data were collected via face-to-face interviews with a professional interviewer using the data collection form that included demographic information, tobacco use, physical activity, hypertension, dyslipidemia, body mass index (BMI), other diseases status, occupational exposure status, presence in cities with air pollution, aquatic food consumption pattern, and supplements status such as vitamin D and omega3. Physical activity scores were calculated using the short version of the International Physical Activity Questionnaire (IPAQ). Subsequently, the scores were classified into three levels (high [HEAP active], moderate [minimally active], and low activity [inactive])^31^. According to NCEP ATP-III hyperlipidemia criteria^32^, the participants with dyslipidemia were identified. In addition, participants with hypertension were identified based on WHO criteria^33^ or on a history of consuming antihypertensive drugs.

### Chemical analysis of PCBs (exposure assessment)

#### Sample collection

The PCBs serum concentration was measured to describe the environmental exposure in this cohort study. To evaluate the exposure level of 181 participants to PCBs, their blood serum samples, stored at − 80 °C for the past 10 years, were utilized. These serum samples were collected and preserved at the end of phase II of the PIB-MONICA-S in 2009. The concentration of nine PCB congeners was determined based on the International Union of Pure and Applied Chemistry (IUPAC) nomination, including 28, 52, 99, 101, 114, 118, 138, 153, and 180. Then, the PCBs exposure was categorized into three clusters of total PCB concentration (ΣPCB: the sum of the nine studied PCB congeners), NDL-PCBs concentration (the sum of seven PCB congeners: 28, 52, 99, 101, 138, 153, and 180), and DL-PCBs concentration (the sum of two PCB congeners: 114, and 118).

#### Serum extraction

The laboratory extraction of PCBs was conducted in a certified private laboratory accredited by the Tehran University of Medical Sciences, employing solid-phase extraction (SPE) as the extraction technique. The laboratory extraction of PCBs was conducted in a certified private laboratory accredited by the Tehran University of Medical Sciences, employing solid phase extraction (SPE) as the extraction technique. The sample concentration and analytical procedure were guided by the methodology described in the study by Lin et al.^34^.

Solid-phase extraction, a modern extraction technique utilized in this study, encompasses four steps: conditioning (to prepare the stationary phase), sample application (to add the sample into a column containing the stationary phase), washing (to wash the column with a suitable solvent to remove interfering substances), and finally elution (to wash the column with a suitable solvent for analyte extraction). The procedure employed a vacuum manifold, nitrogen gas, carbon-bonded cartridges, and a glass connector for solvent concentration and drying. Also, Dichloromethane was used for eluting the analytes under a vacuum following SPE conditioning steps.

#### Gas chromatographic conditions

The PCBs congeners were analyzed and measured using an Agilent 6890–5973 gas chromatography-mass spectrometry (GC–MS) equipped with an HP-5MS column (0.25 µm film thickness, 0.25 mm, i.d., 30 m). The initial temperature of the GC oven column was 90 °C, maintained for one minute, then increased to 150 °C at a rate of 50 °C/min for one minute, and finally increased rapidly to 330 °C at a rate of 8 °C/min, where it was held constant for three minutes. The injector and detector temperatures were adjusted to 250 and 150 °C, respectively. Helium was applied as the carrier gas at a fixed flow rate of 1.0 ml/min, and the transmission line temperature was set at 280 °C. This method enabled the detection of desired analytes in nanograms per liter.

#### Validation procedure (quality assurance/quality control (QA/QC))

A calibration curve was plotted with concentrations of 0.3, 1.5, 5, 15, 25, and 50 ng/L for the mixture of targeted PCB analytes (standard solutions). Linear regression was applied to plot the calibration curve area against concentration. Pearson correlation coefficient was calculated and obtained from 0.96 to 0.99 for various analytes. Moreover, qualitative identification of analytes was done based on the retention time (RT), ion spectrum, and ion ratio. The filament was turned off during the initial 2 min of detection to prevent solvent interference. Hexachlorobenzene used as the internal standard with a concentration of 0.1 μg/liter, which was added to the samples after dissolving in methanol. To check the accuracy of the quantitative assessment, within-day and between-day precision was evaluated in triplicate over three separate days for low, medium, and high standard PCB analyte concentrations (0.5, 7.5, and 35 ng/l). The limit of detection (LOD) and the limit of quantification (LOQ) were obtained equal to 0.1 and 0.3 ng/L, respectively, for each measured congener.

#### Identification and quantification of PCBs

PCB congener concentrations were measured based on a wet weight basis. Due to their lipophilic nature and tendency to associate with blood lipids, a concentration on a lipid-adjusted basis was used^35^. Total serum lipid content was calculated by summing the triglycerides (TG) and total cholesterol (TC) according to the Phillips formula^36^, with values expressed as nanograms or picograms per gram lipid (ng/g lipid or pg/g lipid).

### Statistical analysis

The normality of the data is determined using the Kolmogorov–Smirnov test and the histogram. Homogeneity of variance was assessed using Levene’s Test for Equality of Variances. Data were examined using descriptive analysis, which included the mean (SD), median (interquartile range (IQR)), and frequencies (percentages). The relationship between quantitative variables and the dependent variable was examined using independent t-tests, while chi-square tests were used for categorical variables. Simple logistic regression models tested between type 2 diabetes and demographic, anthropometric, biochemistry characteristics, diet and supplement information of participants, and other diseases separately to identify the potential confounders. Finally, age, waist circumference, family history of diabetes, and dyslipidemia were considered confounding variables. Due to a relatively high loss to follow-up, odds ratio (OR) approach were used instead of relative risk (RR). Multiple logistic regression model was used to evaluate the relationship between PCB serum concentrations with type 2 diabetes. To control the effect of confounders, other covariates (age, waist circumference, family history of diabetes, and dyslipidemia) were included in the model. The obtained data from multiple logistic regression models were demonstrated using OR with confidence interval (95% CI). The Hosmer–Lemeshow test was used to check the goodness-of-fit for the regression model. The model with the highest steps has the best fitness. The level of statistical significance in this analysis was 0.05. In addition, if the reported concentration of the PCB congeners was within the limit of detection or below, half of the detection limit was recorded instead (LOD/2). IBM SPSS Statistics 19 was used for all statistical analysis.

### Ethical considerations

The study protocol and all procedures were approved by the Tehran University of Medical Sciences Ethics Committee (# IR.TUMS.SPH.REC.1398.321). All participants signed and confirmed the written informed consent. Moreover, Participation in the study was voluntary.

## Results

### Baseline characteristics

**Figure 1:**
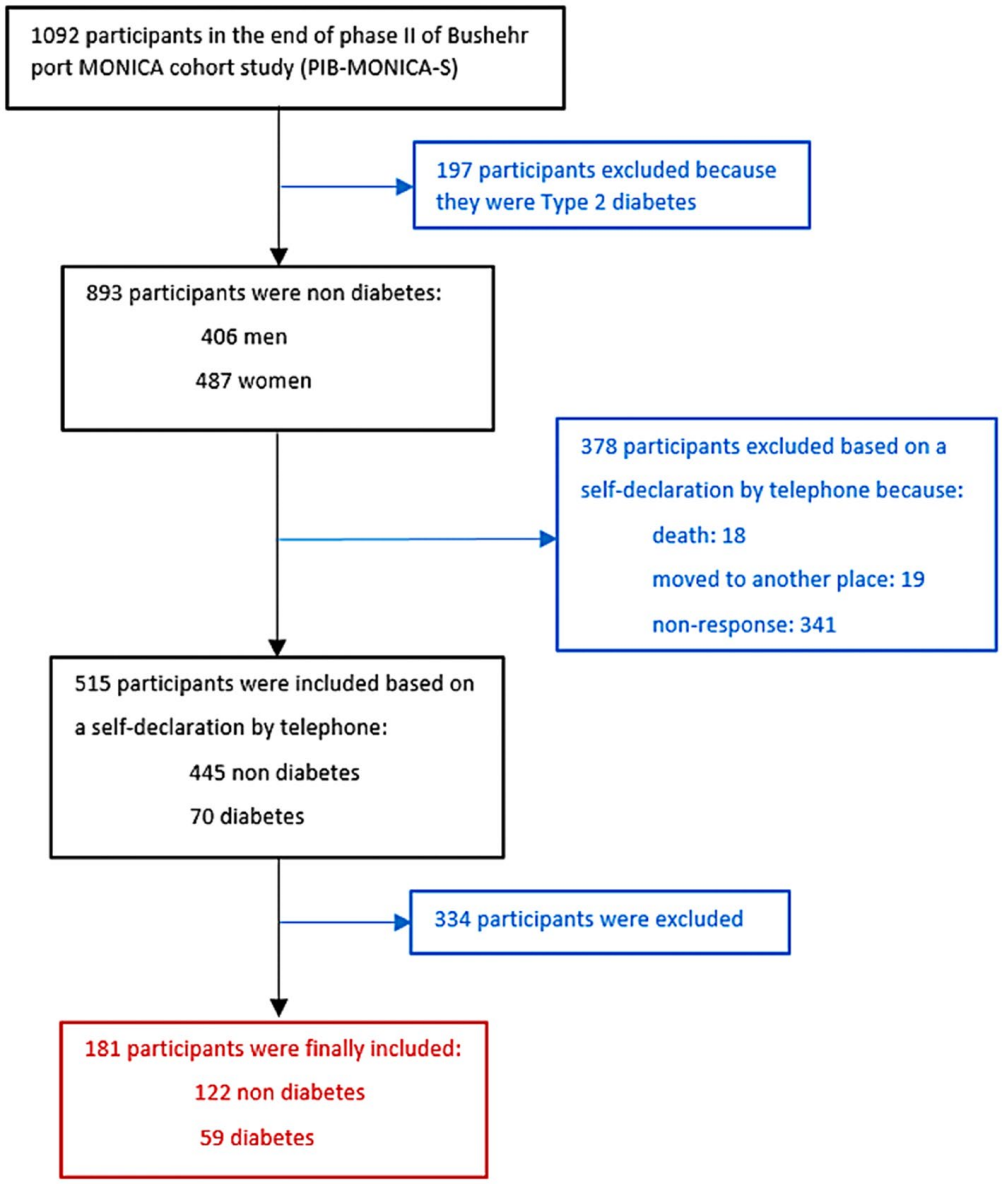
Selection participants’ flow diagram.

We studied a total of 181 participants during ten years of follow-up (Fig. 1), of whom 59 (32.60%) were categorized as having type 2 diabetes; most diabetic participants were females (31 of 59 [52.50%]), and 54 of 59 [91.50%] were married. The mean (SD) age of participants was 54.67 (8.9) years, significantly higher in diabetic than non-diabetic participants (*p* < 0.05). Other characteristics of participants including clinical and laboratory details during follow-up, are summarized in Table 1. Participants at risk for type 2 diabetes were more likely to be overweight or obese (19 of 59 [32.20%] or 26 of 59 [44.06%], respectively). In total, a significant portion of the cohort participants had hypertension (87 of 181 [48.10%]), dyslipidemia (121 of 181 [66.90%]), vitamin D insufficiency and deficiency (127 of 181 [70.16%]), and low physical activity (145 of 181 [80.11%]). These parameters, as well as tobacco use, aquatic diet (consumption of fish and shrimp), and occupational exposure, were less in diabetic compared to non-diabetic participants (*p* > 0.05) (Table 1).

**Table 1:** General characteristics of the study participants.

Parameters	Total (n = 181)	Type 2 diabetes mellitus		*p*-Value
		Yes (n = 59)	No (n = 122)	
Age, mean (SD), years	54.67 ± 8.90	58.64 ± 8.05	52.75 ± 8.68	< 0.001^a^
Weight, mean (SD), kg	77.35 ± 13.96	77.06 ± 13.30	77.48 ± 14.32	
Waist circumference, mean (SD), cm	100.62 ± 9.58	102.08 ± 9.42	99.92 ± 9.62	0.158^a^
Hip circumference, mean (SD), cm	109.56 ± 11.00	109. 00 ± 9.20	109.82 ± 11.79	0.642
Triglyceride (TG), mean (SD), mg/dl	153.87 ± 79.29	165.76 ± 83.93	148.11 ± 76.63	
Total Cholesterol (TC), mean (SD), mg/dl	182.36 ± 40.58	173.08 ± 42.63	186.85 ± 38.95	
HDL Cholesterol, mean (SD), mg/dl	50.13 ± 11.42	49.83 ± 11.46	50.27 ± 11.44	
LDL Cholesterol, mean (SD), mg/dl	101.46 ± 35.04	90.10 ± 32.40	106.95 ± 35.07	
Total serum lipid, mean (SD), mg/dl	568.45 ± 140.47	559.30 ± 158.50	572.90 ± 131.30	0.541
Systolic blood pressure, mean (SD), mmHg	126.25 ± 17.64	132.23 ± 18.94	123.35 ± 16.28	
Diastolic blood pressure, mean (SD), mmHg	80.90 ± 9.46	81.05 ± 8.92	80.83 ± 9.74	
FBS, mean (SD), mg/dl	105.85 ± 30.70	134.25 ± 38.42	92.12 ± 10.53	
Sex, No. (%)				0.828
Male	88 (48.60%)	28 (15.50%)	60 (33.10%)	
Female	93 (51.40%)	31 (17.10%)	62 (34.30%)	
Marital status, No. (%)				0.222
Married	165 (91.10%)	54 (29.80%)	111 (61.30%)	
Single	7 (3.90%)	1 (0.60%)	6 (3.30%)	
Other	9 (5.00%)	4 (2.20%)	5 (2.80%)	
Education, No. (%)				0.211
Academic	45 (24.90%)	13 (7.20%)	32 (17.70%)	
High school	73 (40.30%)	21 (11.60%)	52 (28.70%)	
Guidance school	33 (18.20%)	14 (7.70%)	19 (10.50%)	
Primary school	22 (12.20%)	7 (3.90%)	15 (8.30%)	
Illiterate	8 (4.40%)	4 (2.20%)	4 (2.20%)	
Tobacco Use, No. (%)				0.926
Never	110 (60.80%)	34 (18.80%)	76 (42. 00%)	
Former	25 (13.80%)	12 (6.60%)	13 (7.20%)	
Second-hand user	23 (12.70%)	6 (3.30%)	17 (9.40%)	
Current	23 (12.70%)	7 (3.90%)	16 (8.80%)	
BMI, No. (%)				0.332
Under weight (< 18.5)	3 (1.70%)	0 (0.00%)	3 (1.70%)	
Normal weight (18.5–24.9)	37 (20.40%)	12 (6.60%)	25 (13.80%)	
Over weight (25–29.9)	65 (35.90%)	19 (10.50%)	46 (25.40%)	
Obesity (30–39.9)	70 (38.70%)	26 (14.40%)	44 (24.30%)	
Severe obesity (≥ 40)	6 (3.30%)	2 (1.10%)	4 (2.20%)	
Hypertension, No. (%)				0.007^a^
Yes	87 (48.10%)	37 (20.40%)	50 (27.60%)	
No	94 (51.90%)	22 (12.20%)	72 (39.80%)	
Dyslipidemia, No. (%)				0.6
Yes	121 (66.90%)	41 (22.70%)	80 (44.20%)	
No	60 (33.10%)	18 (9.90%)	42 (23.20%)	
Vitamin D status, No. (%)				0.592
Deficient (< 20 mg/dl)	70 (38.70%)	22 (12.20%)	48 (26.50%)	
Insufficient (20–30 mg/dl)	57 (31.50%)	23 (12.70%)	34 (18.80%)	
Sufficient (≥ 30 mg/dl)	54 (29.80%)	14 (7.70%)	40 (22.10%)	
Physical activity (IPAQ), No. (%)				0.921
Low (inactive)	145 (80.10%)	46 (25.40%)	99 (54.70%)	
Moderate (minimally active)	30 (16.60%)	12 (6.60%)	18 (9.90%)	
High (HEAP active)	6 (3.30%)	1 (0.60%)	5 (2.80%)	
Family history of being diabetes, No. (%)				< 0.001^a^
Yes	81 (44.80%)	41 (22.70%)	40 (22.10%)	
No	98 (54.10%)	17 (9.40%)	81 (44.80%)	
Unknown	2 (1.10%)	1 (0.60%)	1 (0.60%)	
Having the occupational exposure, No. (%)	8 (4.40%)	2 (1.10%)	6 (3.30%)	0.641

*HDL* high-density lipoprotein, *LDL* low-density lipoprotein, *SD* standard deviation, *BMI* body mass index, *FBS* fasting blood sugar, *IPAQ* international physical activity questionnaire.

^a^*p* < 0.2: for selecting covariates by simple logistic regression

### Chemical concentration in participants’ blood

**Figure 2:**
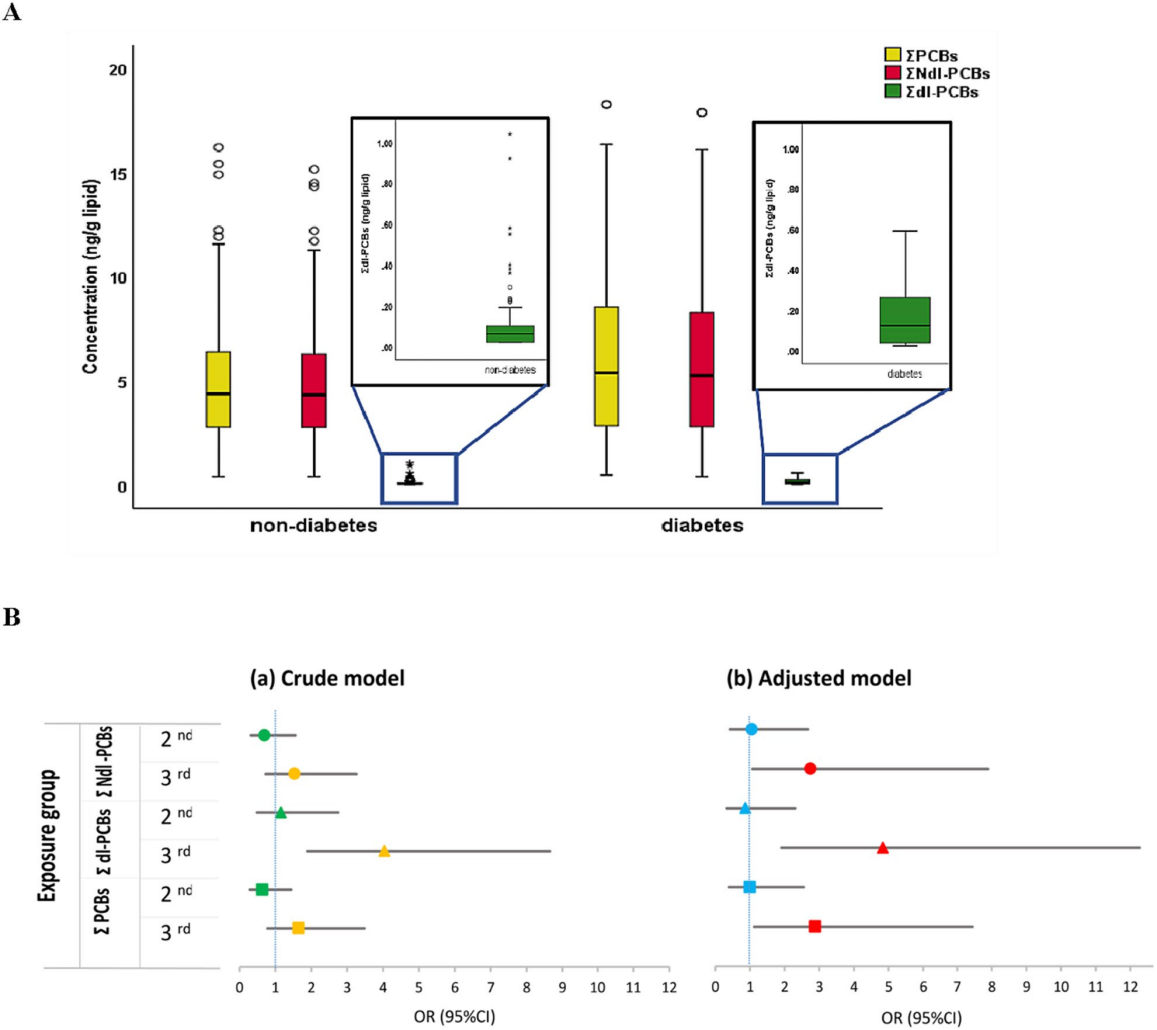
(**A**) Mean serum concentrations of PCBs in diabetes and non-diabetes participants. (**B**) Forest Plot of Multiple logistic regression models for 10-year follow-up between PCBs exposure and type 2 diabetes. Note: CI: confidence interval; OR: odds ratio; ΣPCBs (total Polychlorinated biphenyls); ΣNdl-PCBs (Non-dioxin-like-PCBs); Σdl-PCBs (dioxin-like- PCBs).

Altogether, 181 serum samples from the beginning of the follow-up period (in 2009) were analyzed to measure the concentration of PCBs. The lipid-adjusted serum concentrations of ΣNdl-PCBs, Σdl-PCBs, and ΣPCBs were significantly higher in the diabetes group (*p* < 0.05) (Table 2, Fig. 2A). PCB congeners 153 and 138 exhibited the highest concentrations. Also, they were reported with approximately equal proportions between both groups (PCB138: 40% in diabetes vs. 40.50% in non-diabetes and for PCB153: 50.50% in diabetes vs. 52.30% in non-diabetes). Except for PCB 138 and 153, all tested congeners had lower values than the detection limit in some samples. The concentration of total PCBs was detected in the serum of all participants so that the concentration of any sample was not reported as zero.

**Table 2:** Distribution of the PCBs serum concentration of the study population (n = 181).

Parameters	Mean ± SD	Range	Median	IQR
ΣNdl-PCBs, ng/g lipid	4.708 ± 2.890	0.35–15.34	4.07	2.47–5.97
PCB 28, ng/g lipid	0.063 ± 0.155	0.01–1.18	0.01^b^	0.01–0.01
PCB 52, ng/g lipid	0.038 ± 0.071	0.01–0.48	0.01^b^	0.01–0.01
PCB 99, ng/g lipid	0.051 ± 0.053	0.01–0.29	0.02^a^	0.01–0.09
PCB 101, ng/g lipid	0.025 ± 0.040	0.01–0.32	0.01^b^	0.01–0.01
PCB 138, ng/g lipid	1.937 ± 1.680	0.09–7.50	1.68	1.04–2.62
PCB 153, ng/g lipid	2.486 ± 1.438	0.15–8.14	2.20	1.43–3.22
PCB 180, ng/g lipid	0.117 ± 0.144	0.01–0.92	0.08	0.01–0.15
Σdl-PCBs, ng/g lipid	0.116 ± 0.150	0.02–1.04	0.07	0.02–0.13
PCB 114, ng/g lipid	0.063 ± 0.104	0.01–0.66	0.02^a^	0.01–0.08
PCB 118, ng/g lipid	0.042 ± 0.042	0.01–0.22	0.02^a^	0.01–0.07
Σdl-PCBs, pg/g lipid	116.685 ± 150.399	20.00–1040.00	70.00	20.00–150.00
ΣPCBs, ng/g lipid	4.810 ± 2.978	0.37–15.68	4.08	2.51–6.15

*CI* confidence interval, *OR* odds ratio, *ΣPCBs* total polychlorinated biphenyls, *ΣNdl-PCBs* non-dioxin-like-PCBs, *Σdl-PCBs* dioxin-like-PCBs, *IQR* interquartile range.

^a^The numbers reported are the closest to the 50th percentile, and they were considered as an estimation of the median.

^b^More than 70% of the samples were less than the detection limit, so the median value set half of the LOD.

### Regression models

The logistic regression models for PCBs are shown in Table 3 and Fig. 2B. Table 3 depicts odds ratios (ORs) for single (crude model) and multivariable (adjusted model) logistic regression of three PCBs clusters. The final models included variables with a *p*-value < 0.20 in univariate analysis. After adjusting for age, waist circumference, family history of diabetes, and dyslipidemia, an increased odds ratios (ORs) observed for ΣNdl-PCBs, Σdl-PCBs, and ΣPCBs compared to the crude models. Specifically, multivariable analysis revealed that a one-tertile increase (increasing from 33rd centile to 67th centile) in Σ non-dioxin-like-PCBs (OR 2.749, 95% CI 1.066–7.089), Σ dioxin-like-PCBs (OR 4.842, 95% CI 1.911–12.269), and Σ PCBs (OR 2.887, 95% CI 1.120–7.441) significantly associated with an increased risk of type 2 diabetes (*p* < 0.05). The strongest association was obtained for dioxin-like PCBs (OR 4.842; 95% CI 1.911–12.269; *p* = 0.001). According to the Hosmer–Lemeshow test, all adjusted models demonstrated the best fitness (*p* > 0.05).

**Table 3:** Multiple logistic regression analysis between type 2 diabetes and PCBs serum concentration.

	PCBs tertiles	Crude model		Adjusted model^a^		
		OR (95% CI)	*p*-Value^b^	OR (95% CI)	*p*-Value^b^	Hosmer–Lemeshow *p*-Value^c^
ΣNdl-PCBs, ng/g lipid	1st	Reference		Reference		0.250
	2nd	0.704 (0.317–1.562)	0.388	1.060 (0.420–2.671)	0.902	
	3rd	1.541 (0.730–3.256)	0.257	2.749 (1.066–7.089)	0.036	
Σdl-PCBs, pg/g lipid	1st	Reference		Reference		0.192
	2nd	1.158 (0.488–2.748)	0.740	0.864 (0.323–2.310)	0.770	
	3rd	4.046 (1.891–8.656)	0.000	4.842 (1.911–12.269)	0.001	
ΣPCBs, ng/g lipid	1st	Reference		Reference		0.841
	2nd	0.643 (0.287–1.441)	0.283	1.004 (0.395–2.550)	0.993	
	3rd	1.650 (0.783–3.480)	0.188	2.887 (1.120–7.441)	0.028	

*OR* odds ratio, *CI* confidence interval, *ΣPCBs* total polychlorinated biphenyls, *ΣNdl-PCBs* non-dioxin-like-PCBs, *Σdl-PCBs* dioxin-like-PCBs.

^a^The models were adjusted for age, waist circumference, family history of diabetes, and dyslipidemia.

^b^*p* < 0.05 is significant.

^c^*p* > 0.05: the model is fit.

## Discussion

In this study we aimed to assess the relationship between the serum PCB levels in individuals with type 2 diabetes and those without. This study utilized the high-quality MONICA cohort data with a ten-year follow-up period, incorporating individuals without diabetes at baseline.

Based on this study result, the detected serum PCB concentrations ranged from 0.37 to 15.68 ng/g lipid, with a median value of 4.08 ng/g lipid (interquartile range [IQR] 2.51–6.15). Also, all serum samples exhibited some level of PCB concentration. When compared with findings from global studies, the results revealed lower concentrations than those reported in Pakistan^37^, China^38^, and Japan^39^. These relatively low levels suggest a lesser exposure of the study population to PCBs; however, these minimal concentrations do not diminish its importance. In this regard, scientists also pointed out that exposure to POPs, even at low concentrations, can have significant human health outcomes, including an elevated risk of diabetes^5,40^. Neglecting the population groups with lower exposure risks, compared to those with occupational or accidental exposure, might mask or overlook potential hazards^40^. On the other hand, the results are within the range of PCB concentration detected on occupational exposure groups in Tehran study^41^. Interestingly, the findings indicated higher concentrations than a study involving pregnant women in Tehran, likely attributable to Bushehr’s greater consumption of fish and aquatic foods compared to the national average^42^. Food sources are estimated to account for approximately 90% of PCB exposure portion in Iran, as reported by Ravanipour et al.^43^. Also, PCB153 and PCB138 were detected in all serum samples (with a detection rate of 100%). Similarly, some studies reported these congeners as the most abundant congeners^15,38^. It is generally accepted that the three constituents of PCBs (153 + 138 + 180), on average, about 61% of the PCB burden in the human body^44^.

This study analyzed nine main congeners, categorized into dioxin-like and non-dioxin-like PCBs, and included in the models after adjusting for confounders. The literature displays variability in the number and types of congeners analyzed and their classification into varied subcategories for assessing their association with type 2 diabetes. Some studies have focused on individual congeners (e.g., PCB 153)^17^ or a combination thereof, but most aggregate them into the total PCBs (ΣPCBs) metric^5,18^. Accordingly, the number of congeners used to calculate the total PCBs varied widely. The Stockholm Convention recommends measuring six to seven indicator PCBs (PCB- 28, 52, 101, 138, 153, 180, and 118) due to their higher concentration in food, the environment, or human tissues/fluids^8^. These noteworthy methodological differences may influence the observed relationship with type 2 diabetes.

A significant positive correlation was identified between total PCBs, non-dioxin-like PCBs, and dioxin-like PCBs with type 2 diabetes; the strongest association was observed with dioxin-like PCBs in this study. While some studies support the findings, others report no significant correlation. Airaksinen et al. (2011) confirm a positive association between type 2 diabetes and exposure to PCB153 in a general urban population of the Helsinki Birth Cohort Study^17^. Philibert et al.^18^ also reported a positive association between total PCBs and type 2 diabetes in 101 Canadian participants. In contrast, Jorgensen et al.^16^ in Greenland examined the relationship between 13 of the most common PCBs after adjusting for age, sex, waist circumference, hereditary history, smoking, alcohol consumption, and education level, but no correlation was found. Also, Zani et al.^15^ found no association between total PCBs and diabetes in their study of an urban area in northern Italy where a PCB chemical plant had previously caused severe contamination. Similarly, in a cohort study by Magliano et al.^2^ with nine years of follow-up in France, no association was found between exposure to ΣPCB and diabetes. Although most of these studies have been performed as a case–control or cohort study, the results of meta-analysis studies obtained from the aggregation of such articles have not shown much certainty in their conclusions. These discrepancies underscore the complexity of determining the impact of PCB exposure on diabetes risk. A meta-analysis study conducted by Tang et al.^45^ showed a possible association between PCB153 and type 2 diabetes. The need to conduct smaller studies with more inconsistent results is emphasized in future studies in order to minimize the potential impact of publication bias. Conversely, a meta-analysis study conducted by Song et al.^24^ emphasize the urgent need to investigate the relationship of endocrine-disrupting chemicals (EDCs) with diabetes risk, mainly through large prospective studies.

Moreover, today, the main mechanism of PCBs association with diabetes is controversial and still unclear. Various in vitro and in vivo studies have addressed this issue and mentioned some possible mechanisms, including mitochondrial dysfunction, endocrine disruption (through depletion of pancreatic β cells), and the effect on glucose metabolism^46^. In addition, the various toxic effects of PCBs are related to the chlorine site on the phenyl ring. The high ability of some PCB congeners for binding to aryl hydrocarbon receptor (AhR) causes toxicological properties similar to polychlorinated dibenzo-p-dioxins (PCDD) and places them in the dl-PCBs category^8^. Dioxin-like PCBs, known for their binding affinity to the aryl hydrocarbon receptor (AhR), may impair mitochondrial function via the AhR pathway and lead to diabetes^46^. Although the study did not directly investigate these mechanisms, the strong association with dioxin-like PCBs suggests a potential role for diabetes, which may be due to the mentioned mechanisms.

### Strengths and limitations

To the best of our knowledge, this represents the first historical cohort study to explore the association between PCBs and type 2 diabetes in Iran. Prior investigations assessing the relationship between type 2 diabetes and environmental risks of PCBs encountered some limitations, such as conflicting results, the type of studied population (often not representative of the general population), a limited number of diabetes samples, and inadequate consideration of background or confounding variables. In this study, we tried to reduce limitations by considering the level of occupational exposure, diet, underlying diseases of the participants, and the type of population (via selecting a sample from the general population). The main strengths of the present study are the increase in the probability of obtaining cases due to the ten-year follow-up period, which is a relatively long period. Also, diabetes diagnoses were based on fasting blood sugar tests (FBS) rather than solely on self-declaration.

By the way, the study faced some limitations. First, due to the coincidence of the study period with the COVID-19 pandemic, it was not possible to include all diabetes individuals. Second, because of the impossibility of accessing the participants’ medical records, some data for certain variables such as various diseases, the amount of aquatic food consumption, vitamin D, or other supplements was obtained based on their self-declaration in response to retrospective questions. Other limitations of the present study were due to various factors such as unwillingness to participate, death, relocation, or lack of access to participants. Also, as we used several measures of exposure to PCBs and we conducted multiple comparisons in our study, some significant relationships may was not beyond the chance finding and the corrected estimate of *p*-values may be bigger than reported values. This may be a limitation for *p*-values that were not far less than 0.05. For *p*-values that were extremely small, however, this limitation is not of great importance.

## Conclusions

The study support a significant correlation between exposure to total PCBs, dioxin-like and non-dioxin-like PCBs, with type 2 diabetes after adjusting for potential confounders. These findings emphasis the importance and necessity of implementing educational, health, and policy activities to reduce PCBs exposure and prevent this disease. Additional research, including large longitudinal studies designed with samples from various cohort studies within a community (e.g., Iran), is recommended to thoroughly evaluate the effects and mechanisms by which PCBs influence type 2 diabetes, especially considering all the confounding factors of the disease. Such studies, with considering all potential confounders, enhance comprehensively the understanding of the findings of this study. Furthermore, the findings should motivate further research into the simultaneous examination of PCBs exposures and other reported environmental causal factors (e.g., Cadmium, PAHs, etc.) suspected of contributing to type 2 diabetes. Identifying and addressing the root causes and risk factors associated with the disease can lead to the development of more effective prevention and management strategies. Lastly, Bushehr is a coastal and industrial city, with a hot and humid climate, and a special food culture, so that fish and shrimp are the main food of the citizens; it is recommended to consider these issues in generalizing the findings of this study to other societies.

## Data Availability

The data are not publicly available but may be accessed through the corresponding author on reasonable request.

## References

[1] Ogurtsova K (2017). IDF diabetes Atlas: Global estimates for the prevalence of diabetes for 2015 and 2040. Diabetes Res. Clin. Pract..

[2] Magliano DJ (2021). Exposure to persistent organic pollutants and the risk of type 2 diabetes: A case-cohort study. Diabetes Metab..

[3] WHO. Global Health Estimates: Life expectancy and leading causes of death and disability. https://www.who.int/data/gho/data/themes/mortality-and-global-health-estimates (2022).

[4] WHO. Diabetes. *13 April 2021* https://www.who.int/health-topics/diabetes#tab=tab_1 (2021).

[5] Arrebola JP (2013). Adipose tissue concentrations of persistent organic pollutants and prevalence of type 2 diabetes in adults from Southern Spain. Environ. Res..

[6] Bellou V, Belbasis L, Tzoulaki I, Evangelou E (2018). Risk factors for type 2 diabetes mellitus: An exposure-wide umbrella review of meta-analyses. PLoS One.

[7] Manolio TA (2009). Finding the missing heritability of complex diseases. Nature.

[8] WHO (2016). Safety Evaluation of Certain Food Additives and Contaminants, Supplement 1: Non-Dioxin-Like Polychlorinated Biphenyls/Prepared by the Eightieth Meeting of the Joint FAO/WHO Expert Committee on Food Additives (JECFA).

[9] Carpenter DO (2006). Polychlorinated biphenyls (PCBs): Routes of exposure and effects on human health. Rev. Environ. Health.

[10] UNEP. Stockholm Convention On Persistent Organic Pollutants (POPS), Revised In 2019. *United Nations* http://chm.pops.int/TheConvention/Overview/TextoftheConvention/tabid/2232/Default.aspx (2020).

[11] Malisch R, Kotz A (2014). Dioxins and PCBs in feed and food: Review from European perspective. Sci. Total Environ..

[12] Ali N (2016). Organohalogenated contaminants in type 2 diabetic serum from Jeddah, Saudi Arabia. Environ. Pollut..

[13] UNEP. Persistent Organic Pollutants (POPs) and Pesticides. https://www.unep.org/cep/persistent-organic-pollutants-pops-and-pesticides (2021).

[14] Reddy AVB, Moniruzzaman M, Aminabhavi TM (2019). Polychlorinated biphenyls (PCBs) in the environment: Recent updates on sampling, pretreatment, cleanup technologies and their analysis. Chem. Eng. J..

[15] Zani C (2019). Polychlorinated biphenyl serum levels, thyroid hormones and endocrine and metabolic diseases in people living in a highly polluted area in North Italy: A population-based study. Heliyon.

[16] Jørgensen ME, Borch-Johnsen K, Bjerregaard P (2008). A cross-sectional study of the association between persistent organic pollutants and glucose intolerance among Greenland Inuit. Diabetologia.

[17] Airaksinen R (2011). Association between type 2 diabetes and exposure to persistent organic pollutants. Diabetes Care.

[18] Philibert A, Schwartz H, Mergler D (2009). An exploratory study of diabetes in a first nation community with respect to serum concentrations of *p*, *p*’-DDE and PCBs and fish consumption. Int. J. Environ. Res. Public Health.

[19] Barrios-Rodríguez R (2021). Associations of accumulated selected persistent organic pollutants in adipose tissue with insulin sensitivity and risk of incident type-2 diabetes. Environ. Int..

[20] Raffetti E (2018). Polychlorinated biphenyls (PCBs) exposure and cardiovascular, endocrine and metabolic diseases: A population-based cohort study in a North Italian highly polluted area. Environ. Int..

[21] Codru N (2007). Diabetes in relation to serum levels of polychlorinated biphenyls and chlorinated pesticides in adult Native Americans. Environ. Health Perspect..

[22] Mansouri EH, Reggabi M (2021). Association between type 2 diabetes and exposure to chlorinated persistent organic pollutants in Algeria: A case-control study. Chemosphere.

[23] Lind PM, Lind L (2018). Endocrine-disrupting chemicals and risk of diabetes: An evidence-based review. Diabetologia.

[24] Song Y (2016). Endocrine-disrupting chemicals, risk of type 2 diabetes, and diabetes-related metabolic traits: A systematic review and meta-analysis. J. Diabetes.

[25] Nabipour I (2007). The metabolic syndrome and nonfatal ischemic heart disease; A population-based study. Int. J. Cardiol..

[26] Ostovar A (2014). Hypertension risk and conventional risk factors in a prospective cohort study in Iran: The Persian Gulf Healthy Heart Study. Int. J. Cardiol..

[27] Friedewald WT, Levy RI, Fredrickson DS (1972). Estimation of the concentration of low-density lipoprotein cholesterol in plasma, without use of the preparative ultracentrifuge. Clin. Chem..

[28] CDC. Diabetes Tests: Fasting Blood Sugar Test. *Centers for Disease Control and Prevention* https://www.cdc.gov/diabetes/basics/getting-tested.html (2023).

[29] ADA (2020). 2. Classification and diagnosis of diabetes: Standards of medical care in diabetes—2020. Diabetes Care.

[30] Kasper DL (2018). Harrison’s Principles of Internal Medicine 20/E (Vol. 1 & Vol. 2) (ebook).

[31] IPAQ. Guidelines for Data Processing and Analysis of the International Physical Activity Questionnaire (IPAQ)—Short Form, Version 2.0. April 2004. https://www.physio-pedia.com/images/c/c7/Quidelines_for_interpreting_the_IPAQ.pdf (2004).

[32] Expert Panel on Detection, Evaluation (2001). Executive summary of the third report of the national cholesterol education program (NCEP) expert panel on detection, evaluation, and treatment of high blood cholesterol in adults (adult treatment panel III). Jama.

[33] WHO. *Guideline for the Pharmacological Treatment of Hypertension in Adults* (2021). 34495610

[34] Lin Y, Pessah IN, Puschner B (2013). Simultaneous determination of polybrominated diphenyl ethers and polychlorinated biphenyls by gas chromatography–tandem mass spectrometry in human serum and plasma. Talanta.

[35] Eslami B (2016). Association between serum concentrations of persistent organic pollutants and gestational diabetes mellitus in primiparous women. Environ. Res..

[36] Phillips DL (1989). Chlorinated hydrocarbon levels in human serum: effects of fasting and feeding. Arch. Environ. Contam. Toxicol..

[37] Ali N, Eqani SAMAS, Malik RN, Neels H, Covaci A (2013). Organohalogenated contaminants (OHCs) in human serum of mothers and children from Pakistan with urban and rural residential settings. Sci. Total Environ..

[38] Han X (2020). Associations between the exposure to persistent organic pollutants and type 2 diabetes in East China: A case-control study. Chemosphere.

[39] Inoue K (2006). Levels and concentration ratios of polychlorinated biphenyls and polybrominated diphenyl ethers in serum and breast milk in Japanese mothers. Environ. Health Perspect..

[40] Porta M (2006). Persistent organic pollutants and the burden of diabetes. Lancet (London, England).

[41] Eftekhari S (2018). Association of plasma PCB levels and HbA1c concentration in Iran. J. Occup. Med. Toxicol..

[42] STEPs. Atlas of Non-Communicable Diseases Risk-Factors Surveillance in the Islamic Republic of Iran. https://vizit.report/panel/steps/fa/main.html#/forestLocation (2016).

[43] Ravanipour M, Nabipour I, Yunesian M, Rastkari N, Mahvi AH (2022). Exposure sources of polychlorinated biphenyls (PCBs) and health risk assessment: A systematic review in Iran. Environ. Sci. Pollut. Res..

[44] European Food Safety Authority (EFSA) (2005). Opinion of the scientific panel on contaminants in the food chain [CONTAM] related to the presence of non dioxin-like polychlorinated biphenyls (PCB) in feed and food. EFSA J..

[45] Tang M, Chen K, Yang F, Liu W (2014). Exposure to organochlorine pollutants and type 2 diabetes: A systematic review and meta-analysis. PLoS One.

[46] Yang C, Kong APS, Cai Z, Chung ACK (2017). Persistent organic pollutants as risk factors for obesity and diabetes. Curr. Diabetes Rep..

